# *Helicobacter pylori* Avoids the Critical Activation of NLRP3 Inflammasome-Mediated Production of Oncogenic Mature IL-1β in Human Immune Cells

**DOI:** 10.3390/cancers12040803

**Published:** 2020-03-27

**Authors:** Suneesh Kumar Pachathundikandi, Nicole Blaser, Heiko Bruns, Steffen Backert

**Affiliations:** 1 Department of Biology, Division of Microbiology, Friedrich-Alexander University Erlangen-Nuremberg, Staudtstr. 5, D-91058 Erlangen, Germany; nicole.albrecht@fau.de; 2 Department of Internal Medicine 5, Hematology and Oncology, University Hospital Erlangen, Friedrich-Alexander University, D-91058 Erlangen, Germany; heiko.bruns@uk-erlangen.de

**Keywords:** Inflammasome, NLRP3, *Helicobacter pylori*, Interleukin-1β

## Abstract

*Helicobacter pylori* persistently colonizes the human stomach, and is associated with inflammation-induced gastric cancer. Bacterial crosstalk with the host immune system produces various inflammatory mediators and subsequent reactions in the host, but not bacterial clearance. Interleukin-1β (IL-1β) is implicated in gastric cancer development and certain gene polymorphisms play a role in this scenario. Mature IL-1β production depends on inflammasome activation, and the NLRP3 inflammasome is a major driver in *H. pylori*-infected mice, while recent studies demonstrated the down-regulation of NLRP3 expression in human immune cells, indicating a differential NLRP3 regulation in human vs. mice. In addition to the formation of mature IL-1β or IL-18, inflammasome activation induces pyroptotic death in cells. We demonstrate that *H. pylori* infection indeed upregulated the expression of pro-IL-1β in human immune cells, but secreted only very low amounts of mature IL-1β. However, application of exogenous control activators such as Nigericin or ATP to infected cells readily induced NLRP3 inflammasome formation and secretion of high amounts of mature IL-1β. This suggests that chronic *H. pylori* infection in humans manipulates inflammasome activation and pyroptosis for bacterial persistence. This inflammasome deregulation during *H. pylori* infection, however, is prone to external stimulation by microbial, environmental or host molecules of inflammasome activators for the production of high amounts of mature IL-1β and signaling-mediated gastric tumorigenesis in humans.

## 1. Introduction

*Helicobacter pylori* infection in the global human population is a major health burden in many parts of the world. The advent of antibiotic therapy and improved hygiene protocols drastically reduced the burden in several developed countries in recent decades, but lack of diagnosis and poor hygiene in underdeveloped countries made the condition worse with a high percentage of colonized individuals [1]. A meta-analysis of published data revealed that 44.3% to 60.3% of the global population harbors this bacterium in their stomach [2]. The country-specific prevalence varies from 18.9% in Switzerland to 87.7% in Nigeria [1]. *H. pylori* colonization causes mild gastritis in every colonized individual, however, 10–20% of cases are associated with the development of peptic ulcers, 1–2% develop gastric cancer and <1% cause gastric mucosa-associated lymphoid tissue lymphoma (MALT) [3,4]. *H. pylori* is a bacterial carcinogen recognized by the World Health Organization (WHO) due to the association with gastric cancer and MALT lymphoma [5]. The burden of gastric cancer increases every year and 1,033,701 new cases were reported and 782,685 people died worldwide in 2018 as per data of the International Agency for Research on Cancer (IARC) (http://gco.iarc.fr). Once colonized in childhood, *H. pylori* infection can last for the whole life, if not eradicated by prophylactic measures.

*H. pylori* colonization induces various pattern recognition receptors (PRRs) of the host to produce robust chemokine and cytokine responses through signal transduction events in different cellular systems in the gastric tissue [6,7,8]. This bacterium utilizes many of its virulence factors to overcome the natural barriers in the host and establish colonization. Highly virulent type-I *H. pylori* strains harbor the ~40 kb cytotoxicity associated gene pathogenicity island (*cag*PAI) in their genomes, which codes for a type IV secretion system (T4SS) and the effector protein CagA. T4SS-dependent delivery of CagA leads to phosphorylation by c-Src and c-Abl kinases, and both phosphorylated and non-phosphorylated CagA cause perturbation in signaling, cellular functions, morphology and eventually oncogenesis [9,10,11,12,13]. In contrast, less virulent type-II strains do not encode the *cag*PAI in their genomes. Moreover, type-I strains are generally implicated in the development of associated pathologies due to increased inflammation in the colonized individuals. In addition, *H. pylori* secretes a toxin called vacuolating cytotoxin A (VacA), which causes vacuolation of epithelial cells in culture. *VacA s1-i1-m1* allele-harboring strains showed maximum vacuolization in epithelial barrier dysfunction and thereby are associated with peptic ulcer and gastric cancer [14,15,16]. Multiple receptors are reported for VacA on epithelial cells, however, CD18 is the only receptor identified in immune cells like T cells [16,17]. VacA binding to host receptors induce several signaling events, mitochondrial damage, and apoptotic cell death [18,19,20,21]. The *H. pylori* flagellum helps to travel through the thick gastric mucous layer to interact with the gastric epithelium. The hostile acidic gastric environment is generally neutralized by the activity of *H. pylori* urease and GGT (γ-glutamyl transferase) enzymes. Furthermore, an array of adhesion molecules helps for specific attachment to the epithelium [22,23,24]. The establishment of colonization and PRR responses attract various immune cells, especially a high population of neutrophils to infiltrate into the site of infection [25,26,27]. Furthermore, the immune responses through cellular and soluble mediators lead to the development of adaptive immunity against this bacterium. *H. pylori* elicits strong T cellular and B cellular responses to mainly develop Th1 and Th17 effector cell populations and strong humoral immunity through antibodies, respectively [26,28,29,30,31]. Macrophages were reported to be differentiated into mixed pro-inflammatory M1 and anti-inflammatory M2 populations during *H. pylori* infection [32,33,34]. In addition, *H. pylori* infection causes anti-inflammatory T-regulatory (Treg) cell production to favor suppressed inflammatory reactions and dysregulated gastric microbiota [35,36,37,38].

Interestingly, persistent infection by *H. pylori* is also beneficial for the human host through suppressing other illnesses including asthma, allergies and inflammatory bowel disease [35,37,39,40]. Host gene polymorphisms are known to have a crucial impact in producing different associated pathologies of *H. pylori* infection [41,42,43]. In particular, a specific interleukin-1β (IL-1β) gene polymorphism has prominence and was implicated in hypochlorhydria and gastric cancer development [41,44,45,46]. IL-1β signaling works as an inhibitor of acid secretion and causes gastric atrophy, which provides a hotbed for metaplasia and gastric cancer development. Furthermore, it directly induces the proliferation of gastric carcinoma cells [47,48,49,50]. The secreted IL-1β levels correlated with gastric inflammation and gastric carcinogenesis [51,52,53]. In addition, stomach specific expression of mature human IL-1β in mice developed the stepwise progression of gastric inflammation, dysplasia and gastric cancer and Th1 specific immunity promoted this process [54,55]. However, IL-1β deficiency or IL-1 receptor blockade inhibited these processes [54,55]. In concurrence, lymphocyte-deficient mice expressing IL-1β in the stomach exhibited atrophic gastritis, metaplasia and dysplasia, which supports an independent role of IL-1β in gastric carcinogenesis [54]. *H. pylori*-related MALT lymphomagenesis was reported to be associated with specific intra-tumoral T cell responses. Moreover, translocated CagA mediates B-cell proliferation, leading to lymphomagenesis of MALT lymphomas [56]. In general, the production of mature IL-1β and IL-18 is mediated through the formation of multi-protein scaffolds, called inflammasome and caspase-1 activation [57,58,59], whereas there are some other proteases that can also cleave the pro-forms of these cytokines [60]. Inflammasomes are mainly formed by NLRP1 (Nod-like receptor family, pyrin domain containing 1), NLRP3, NLRC4 (Nod-like receptor family, card domain containing 4), AIM2 (absent in melanoma 2) and Pyrin/TRIM20 (tripartite motif 20) signaling platforms [57,58,59,61]. The NLRP3 inflammasome is the most studied inflammasome, and generally requires two major signaling events for activation, signal-1 and signal-2. The first signal induces the increased expression of components such as NLRP3, ASC (apoptosis associated speck protein), pro-IL-1β, and caspase-1. Finally, inflammasome scaffold formation and activation requires a second signal commonly provided by various microbial, environmental and host molecules such as Nigericin, silica, asbestos, monosodium urate (MSU), ATP or reactive oxygen species (ROS) [62,63,64]. Mice infected with *H. pylori* were reported to activate the NLRP3 inflammasome and secreted mature IL-1β from immune cells through a TLR2/NOD2-dependent mechanism [39,65]. It should be also noted that most of the inflammasome studies on *H. pylori* were done in mice or with isolated mouse dendritic cells (DCs), and identified different virulence factors such as *cag*PAI, VacA, LPS and urease B that are involved in this process [39,65,66]. In addition, chronic *H. pylori* infection in mice regulates inflammasome activation through a MUC1-dependent mechanism [67]. However, very little data exists on inflammasome formation in *H. pylori*-infected human cells and some controversy occurs with regard to the involvement of different virulence factors in the mechanism of activation when compared to mice [39,53,65,66]. In addition to this, we recently found that NLRP3 expression regulation occurred in *H. pylori*-infected human monocytes/macrophages, which was dependent on miR-223-3p and the presence of secreted IL-10 [68]. Consequently, the regulation of the inflammasome by *H. pylori* in mouse versus human cells is not fully understood. Thus, we aimed here to study in more detail the NLRP3 inflammasome formation and secretion of mature IL-1β in *H. pylori*-infected human monocytes/macrophages and resolve some of the conflicting data. We have also reconstructed the NLRP3 inflammasome by overexpression of its components in a non-competent HEK293 epithelial cell line (HEK293-NLRP3-INSOME) and used it for the validation of monocyte/macrophage functions. We found that NLRP3 inflammasome formation and activation not occurred during *H. pylori* infection of human cells, however, which can be surmounted by induction with exogenous second signal activators. This is an important finding that acute infection by *H. pylori* creates a partial situation for NLRP3 inflammasome activation, which may be completed by the release of high molar ATP, monosodium urate (MSU), ROS, exposure to orally passing agents or anything likely to produce high amounts of oncogenic mature IL-1β. These results can partly explain the clearance of *H. pylori* in mice versus chronic infection in humans.

## 2. Results

### 2.1. Different Clinical H. pylori Strains Induce Upregulated Pro-IL-1β Expression in Infected THP1 Monocytes

**Figure 1:**
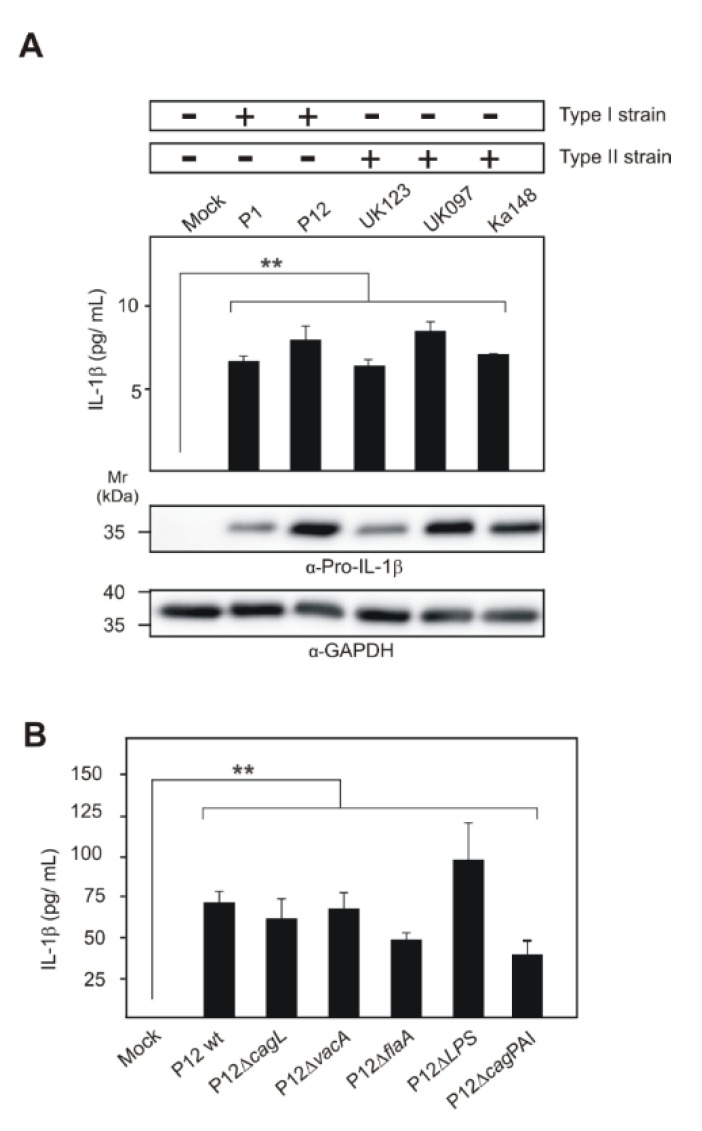
Induced expression of pro-IL-1β in *H. pylori*-infected THP1 monocytes. The first signal of NLRP3 inflammasome activation requires the optimal production of inflammasome components including pro-IL-1β, which is not generally expressed in immune cells without induction. Different wild-type strains including type-I and type-II *H. pylori* were infected for 6 h to analyze the pro-IL-1β expression and secretion (**A**). Furthermore, *H. pylori* P12 wild-type and isogenic mutants were also used to identify the effect on secretion at 24 h of infection with THP1 monocytes (**B**). GAPDH protein immunoblots were used as loading reference. ** *p* ≤ 0.01.

We have previously shown that THP1 monocytes, a commonly used human model cell line for inflammasome activation, constitutively express NLRP3 and pro-caspase-1. Furthermore, NLRP3 expression increased after 6 h of infection with *H. pylori*, but downregulated at a later period [68]. Thus, we used 6 h of infection in the current inflammasome activation experiments unless stated otherwise. The expression of pro-IL-1β is a prerequisite for mature IL-1β production, and its secretion proceeds through activation of the inflammasome. *H. pylori* infection-mediated IL-1β expression and secretion have important roles in pathogenesis. We infected THP1 monocytes with *H. pylori* belonging to the highly virulent type-I (P1 and P12) or less-virulent type-II (UK123, UK097 and Ka148) strains. Both types of *H. pylori* induced the profound expression of pro-IL-1β in THP1 monocytes at 6 h of infection compared to mock-treated control cells (Figure 1A; Supplementary Figure S1). The relative amounts of pro-IL-1β protein expressions were quantified (Supplementary Figure S1). However, cells infected with both types of *H. pylori* secreted very small quantities of mature IL-1β (5–10 pg/mL) as determined by standard ELISA (Figure 1A). Moreover, THP1 monocytes infected with P12 wild-type and isogenic mutants in major virulence factors (*ΔcagL*, *ΔvacA*, *ΔflaA*, *ΔLPS*, *Δcag*PAI) secreted similar amounts of mature IL-1β even after 24 h (Figure 1B), which suggests that these factors are not involved in this process. By comparison with reported studies in mice, DCs infected with *Δcag*PAI and *ΔcagL* mutants of P12 strain showed a significant reduction in IL-1β secretion compared to wild-type bacteria (200–800 pg/mL), and furthermore, priming with *E. coli* LPS drastically increased (above 2000 pg/mL) the IL-1β secretion [39,65]. This shows that *H. pylori*-infected human and mouse cells exhibit significant differences in cleavage of pro-IL-1β and secretion of the mature form. Therefore, our further studies aimed to investigate inflammasome activation in *H. pylori*-infected human cells.

### 2.2. Comparison of Canonical NLRP3 Inflammasome Activation with that of H. pylori-Infected Cells

**Figure 2:**
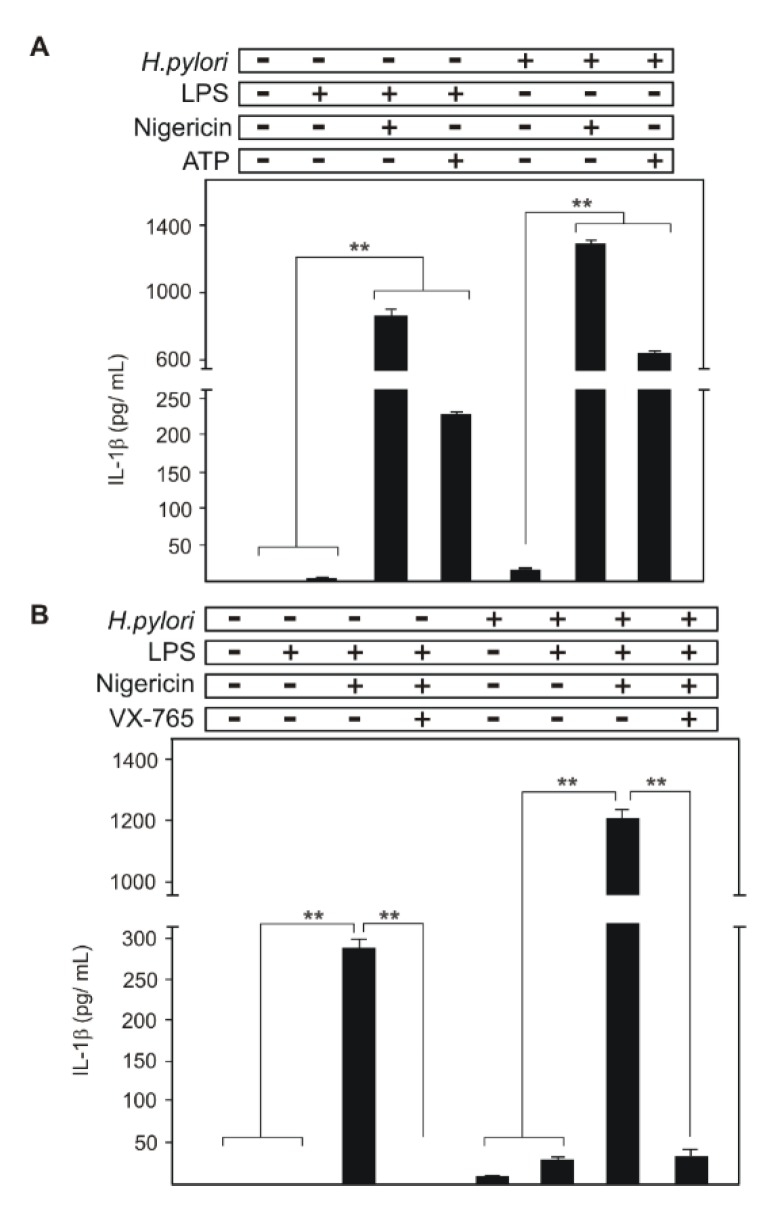
NLRP3 inflammasome activation in THP1 monocytes was compared with *H. pylori* infection at 6 h of treatment. *E. coli* LPS/Nigericin and LPS/ATP were used as signal-1/signal-2 for canonical activation of the NLRP3 inflammasome in THP1 monocytes as described in the text (**A**). *H. pylori* infection secreted low amounts of IL-1β when compared with canonical NLRP3 inflammasome activation-mediated mature IL-1β secretion. Moreover, *E. coli* LPS/*H. pylori* co-treatment showed a corresponding increase in IL-1β secretion but this was not comparable with the inflammasome activation response (**B**). However, *H. pylori*-infected cells treated with signal-2 activators Nigericin or ATP induced the production of a high amount of mature IL-1β secretion (**A**,**B**). The caspase-1 inhibitor VX-765 inhibited all NLRP3 inflammasome driven IL-1β secretion in LPS treated or *H. pylori*-infected cells after activation but infected cells maintained basic secretion (**B**). ** *p* ≤ 0.01.

We next analyzed whether differences exist between canonical NLRP3 inflammasome activation in THP1 monocytes versus *H. pylori* infection. NLRP3 inflammasome formation and activation was carried out by treatment with *E. coli* LPS (as signal-1) followed by addition of 10 µM Nigericin or 5 mM ATP (as signal-2), and determined mature IL-1β production and secretion by ELISA. As expected, *E. coli* LPS-treated cells induced with Nigericin or ATP secreted significantly high amounts of mature IL-1β (Figure 2A). However, cells treated with *E. coli* LPS or *H. pylori* alone secreted no or only small amounts of mature IL-1β (Figure 2A; data not shown). As above, *H. pylori* infection of THP1 monocytes secreted significantly less mature IL-1β, thus we hypothesized that signal-2 for activation is missing upon infection. In this context, we treated *H. pylori*-infected cells with signal-2 activators, and surprisingly, Nigericin or ATP addition significantly increased the secretion of mature IL-1β, which complies with our hypothesis that *H. pylori* lacks the signal-2 for activation of NLRP3 inflammasome in THP1 cells (Figure 2A). This result is also in line with NLRP3 inflammasome activation-mediated secretion in mouse DCs after infection or treatment with *H. pylori* and LPS/ATP, respectively [66]. In fact, this shows interference of this important innate immune response by *H. pylori* in human cells. Moreover, we analyzed *H. pylori*/Nigericin inflammasome activation for the involvement of caspase-1, as other caspases and proteases were also implicated in the cleavage of pro-IL-1β [60]. THP1 monocytes were treated with caspase-1 inhibitor VX-765 (10 µM) 15 min before LPS treatment or infection with *H. pylori*, which followed Nigericin-mediated NLRP3 inflammasome activation. The VX-765 pre-treatment completely abolished caspase-1-dependent mature IL-1β secretion in LPS/Nigericin treated cells (Figure 2B). However, we noted small amounts of IL-1β secretion from VX-765 treated cells exposed to *H. pylori*/Nigericin, which is equivalent to the IL-1β production solely by infection.

### 2.3. Inflammasome Activation in H. pylori-Infected NLRP3 and Caspase-1 Knockout THP1 Monocytes

**Figure 3:**
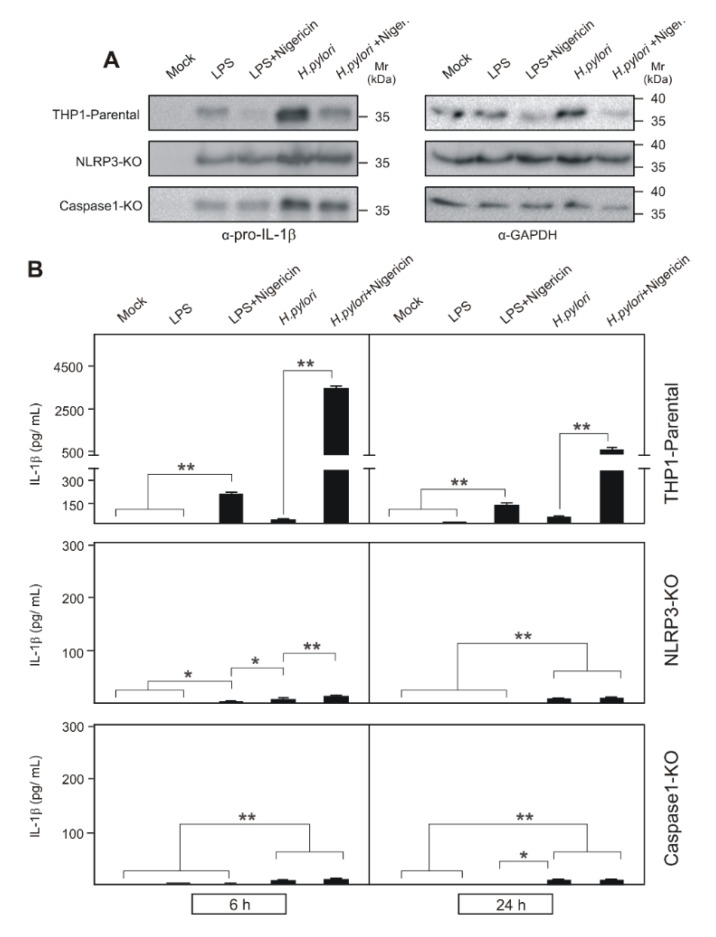
Expression of pro-IL-1β in THP1 parental, THP1 NLRP3-KO and THP1 Caspase-1-KO monocytes after *E. coli* LPS treatment, *H. pylori* infection and NLRP3 inflammasome activation with Nigericin were shown in Western blots in comparison with control GAPDH as loading reference (**A**). GAPDH, a known target for active caspase-1 [69] is also cleaved in THP1 parental cells but not in NLRP3-KO or caspase-1-KO cells after Nigericin treatment, which suggests pyroptotic death (**A**). NLRP3 inflammasome activation is evident with cleavage of pro-IL-1β and secretion of high amount of mature IL-1β from THP1 parental monocytes but not in NLRP3-KO or caspase-1-KO cells after Nigericin treatment, however, both knockout cells secreted basic background secretion after *H. pylori* infection, which again confirms the inflammasome or caspase-1-independent secretion of IL-1β by *H. pylori* (**B**). * *p* ≤ 0.05; ** *p* ≤ 0.01.

To further substantiate our findings, NLRP3 and caspase-1 were knocked-out in THP-1 by CRISPR/Cas9 technology and named as NLRP3-KO or CASP1-KO cells, respectively. LPS-treated and *H. pylori*-infected NLRP3-KO and CASP1-KO THP1 cells were used to investigate inflammasome activation compared to parental control cells. *H. pylori* infection of NLRP3-KO and CASP1-KO cells upregulated pro-IL-1β expression in similar levels observed for parental cells (Figure 3A; Supplementary Figure S2). Active caspase-1 also targets GAPDH [69], being cleaved in THP1 parental cells after Nigericin treatment, while not in NLRP3-KO or caspase-1-KO cells, indicating the stimulation of pyroptotic cell death. Nigericin-induced activation of the NLRP3 inflammasome in LPS-treated NLRP3-KO or CASP1-KO cells did not yield any significant mature IL-1β secretion at 6 h compared to parental cells (Figure 3B). Although, *H. pylori* infection of these knockout cells secreted small amounts of IL-1β as in parental cells, which again confirms that this low secretion is not dependent on NLRP3 inflammasome and caspase-1 activation (Figure 3B). Furthermore, LPS/Nigericin-treated parental cells drastically reduced the mature IL-1β secretion at 24 h when compared with early phase induction, which is implicated for decreased NLRP3 expression in the late phase observed in previous studies [68,70]. *H. pylori*/Nigericin-treated cells also exhibited a similar decrease in mature IL-1β secretion at later periods of infection due to both decreased NLRP3 expression [68] and inflammasome activation. However, infection-induced small amounts of IL-1β secretion gradually increased at a later time point, which again suggests an inflammasome/caspase-1 independent mechanism for this in human THP-1 cells.

### 2.4. Activation of NLRP3 Inflammasome in Human Primary M1 and M2 Macrophages during H. pylori Infection

**Figure 4:**
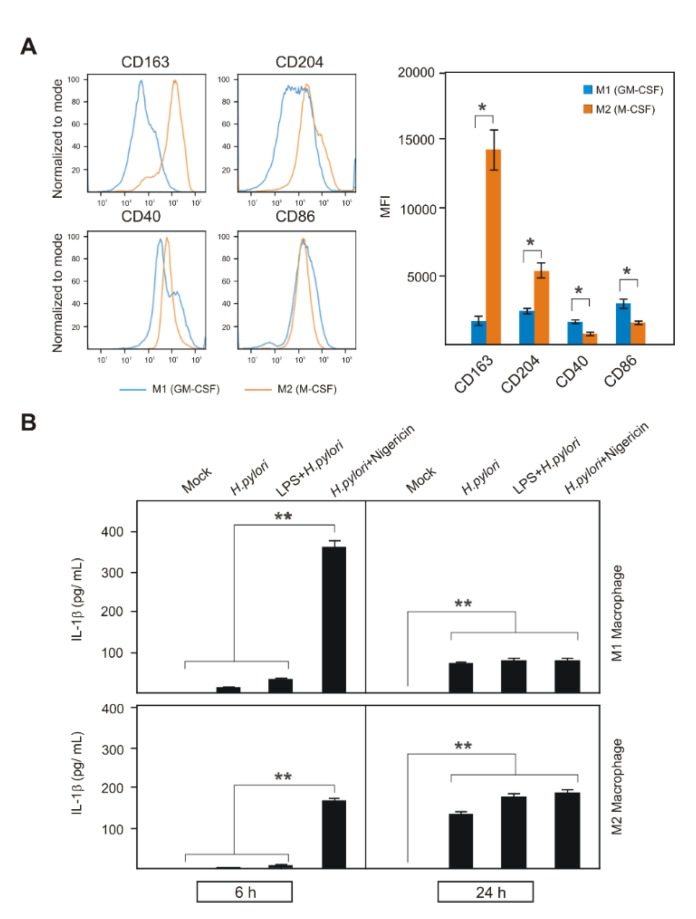
Primary human M1 and M2 macrophages express significantly increased amounts of M1 markers CD40, CD86 and M2 markers CD163, CD204 by flow cytometry (**A**). These M1 and M2 macrophages were infected with *H. pylori*, co-treated with LPS and further treated with Nigericin to analyze NLRP3 inflammasome activation and mature IL-1β secretion (**B**). M1 and M2 macrophages showed a response that was similar to the THP1 monocytes. * *p* ≤ 0.05; ** *p* ≤ 0.01.

Mixed populations of pro- and anti-inflammatory M1 and M2 macrophages, respectively, were reported to be present in the gastric mucosa of *H. pylori*-infected individuals [32,33,34]. To test their importance, peripheral blood monocytes were isolated from three volunteers and differentiated to M1 and M2 macrophages, which was confirmed by flow cytometric analysis of overexpression of M1 markers (CD40 and CD86) and M2 markers (CD163 and CD204) (Figure 4A). These cells were used for studying *H. pylori*-, LPS/*H. pylori*- and *H. pylori*/Nigericin-induced IL-1β expression and secretion. As observed in THP1 cells, *H. pylori*- and LPS/*H. pylori*-infected primary M1 and M2 cells secreted IL-1β, which gradually increased between 6 h to 24 h of infection (Figure 4B). Furthermore, Nigericin-induced NLRP3 inflammasome activation in *H. pylori*-infected M1 and M2 macrophages significantly secreted high amounts of mature IL-1β (Figure 4B). Furthermore, M1 macrophages secreted higher amounts of mature IL-1β in *H. pylori*/Nigericin-treated cells at the early phase, but decreased at the later time point, which again confirms the downregulation of NLRP3 expression in infected primary cells [68]. Moreover, Nigericin-induced NLRP3 activation in infected M2 macrophages significantly increased mature IL-1β secretion at an early time point (Figure 4B). Whereas, this level was maintained at the later time point, but it was not highly significant from infected cells without Nigericin treatment. *H. pylori* infection of M2 macrophages gradually increased significant IL-1β secretion at the later time point, but were not highly responsive to Nigericin treatment, which also supports NLRP3 expression regulation in M2 macrophages Thus, pro- and anti-inflammatory M1 and M2 macrophages, respectively, and the THP1 cell line, exhibited a similar regulation in NLRP3 inflammasome activation-mediated mature IL-1β secretion at the later time point (Figure 4B). Together, *H. pylori* induces the first signal of NLRP3 inflammasome in human primary cells and the THP1 cell line; however, it lacks a proper second signal activation and mature IL-1β secretion.

### 2.5. NLRP3 Inflammasome Characteristic in H. pylori-Infected Cells

**Figure 5:**
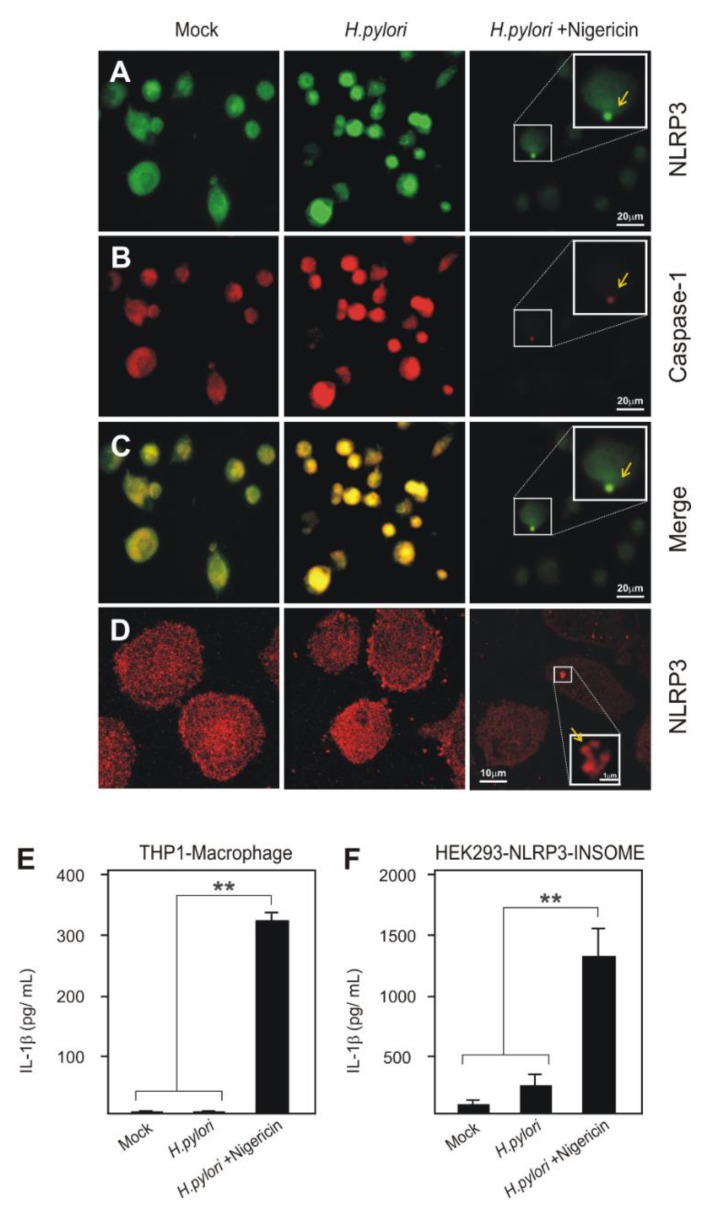
The immuno-fluorescence images of NLRP3, caspase-1 and merged in *H. pylori*-infected, *H. pylori*/Nigericin-treated and mock treated control cells of PMA differentiated THP1 macrophages (**A**–**C**). The NLRP3 inflammasome formation is indicated with yellow arrows. The super resolution STED microscopy of the NLRP3 and inflammasome formation in *H. pylori*-infected, *H. pylori*/Nigericin-treated and mock treated control cells with more clarity showing donut-shaped inflammasome speck (**D**). The mature IL-1β secretion from THP1 macrophages and reconstructed HEK293-NLRP3-INSOME secretion after infection and Nigericin treatment were determined in parallel by ELISA (**E**,**F**). ** *p* ≤ 0.01.

We next infected PMA-differentiated THP1 macrophages with *H. pylori* for investigating NLRP3 inflammasome formation at 6 h. After infection, THP1 macrophages were fixed and stained using immuno-fluorescence labelling against NLRP3 and caspase-1 for visualization by widefield fluorescence microscopy. The careful analysis of *H. pylori*-infected cells and mock control cells did not reveal typical NLRP3 inflammasome speck formation, but a homogenous distribution of the protein. However, the infected cells treated with signal-2 inducer, Nigericin, clearly formed the expected inflammasome speck (Figure 5A). In addition, the labelling for pro-caspase-1 merged at the corresponding spot of the NLRP3 inflammasome speck in *H. pylori/*Nigericin-treated THP1 macrophages, which confirms the close interaction of both of these molecules for active inflammasome formation (Figure 5B,C). The detailed characteristic of NLRP3 inflammasome formed in these cells was further visualized by super resolution STED (stimulated emission depletion) microscopy. As seen in the widefield fluorescence microscope, *H. pylori*-infected, and mock-treated control cells did not show the organization of the inflammasome and cells had a normal size and appearance. After induction with Nigericin in *H. pylori*-infected cells, NLRP3 inflammasome was visualized at super resolution in the characteristic donut shape (Figure 5D) [71], which confirmed the need for the exogenous second signal in NLRP3 inflammasome formation during *H. pylori* infection. 

NLRP3 inflammasome activation in the Nigericin-treated infected (*H. pylori*/Nigericin) cells was confirmed by the significantly high secretion of mature IL-1β (Figure 5E). Next, we transfected inflammasome-deficient HEK293 epithelial cells with eGFP-NLRP3 inflammasome constructs, generating HEK293-NLRP3-INSOME cells, which was previously described for mouse cells and NLRs [72,73]. The basic inflammasome response was not drastically changed in these cells upon infection with *H. pylori*, but Nigericin treatment significantly increased the secretion of mature IL-1β (Figure 5F). These data further confirm the inability of *H. pylori* to establish proper NLRP3 inflammasome functions in human cells.

### 2.6. Reconstructed NLRP3 Inflammasome Speck Induction by a Known Bacterial Activator and H. pylori

**Figure 6:**
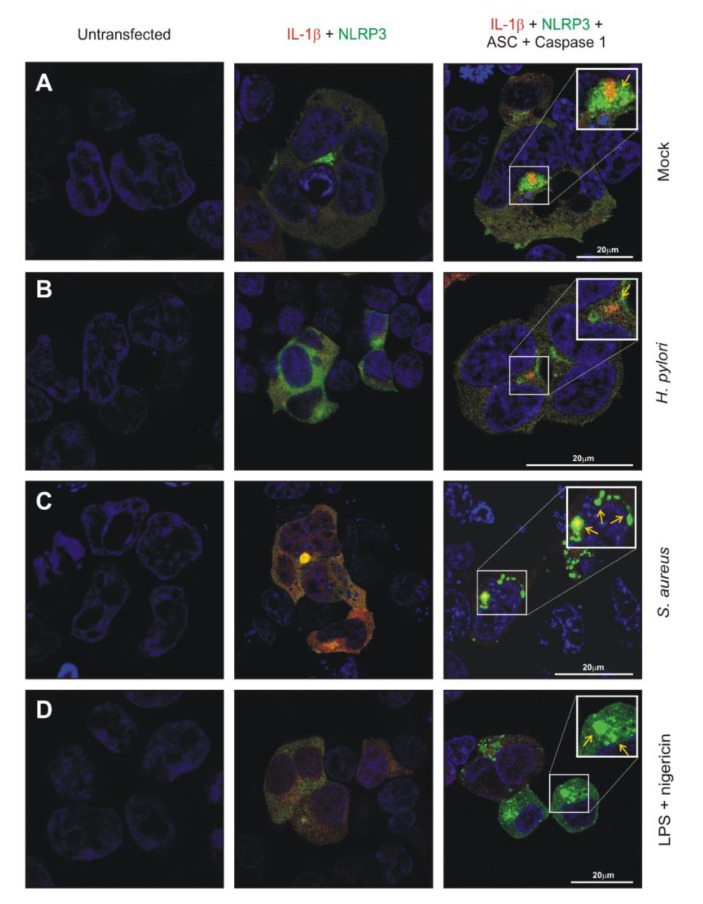
Confocal laser scanning microscopy was used to analyze the reconstructed HEK293-NLRP3-INSOME cells in mock control (**A**), *H. pylori*-infected (**B**), *S. aureus-*infected (**C**) and *E. coli* LPS/Nigericin-treated (**D**). EGFP-NLRP3 and mCherry-pro-IL-1β were visualized using their characteristic colors and NLRP3 inflammasome in various conditions mentioned above were marked with yellow arrows. The respective column heading shows the transfection status of each cell before or after infection or treatment.

HEK293-NLRP3-INSOME cells were used for further analysis on understanding the inflammasome activation dynamics. Activation of NLRP3 inflammasome in HEK293-NLRP3-INSOME cells with the bacterial activator *Staphylococcus aureus* and LPS/Nigericin were compared with that of *H. pylori* infection. The eGFP-NLRP3 and mCherry-pro-IL-1β were visualized in the mock control, LPS/Nigericin treated, *H. pylori*, and *S. aureus* infected HEK293-NLRP3-INSOME cells by confocal laser scanning fluorescence microscopy (CLSM) (Figure 6A–D). Interestingly, *S. aureus-*infected and LPS/Nigericin-treated cells induced more NLRP3 inflammasome specks merged with the mCherry-pro-IL-1β, while *H. pylori*-infected and mock-treated cells maintained similar speck-free features (Figure 6A–D).

### 2.7. Impact of Known H. pylori Virulence Factors

**Figure 7:**
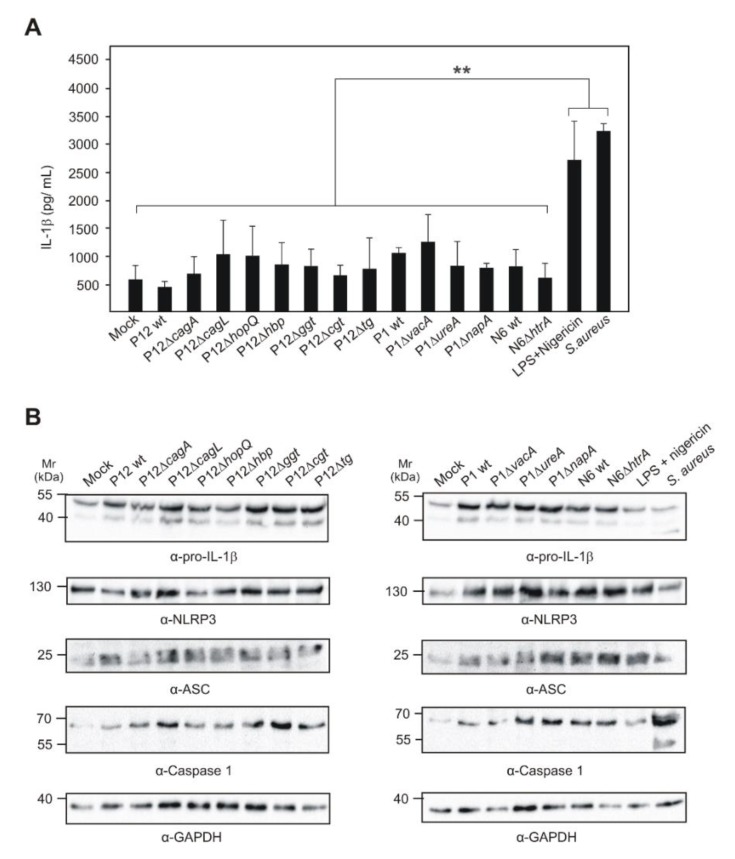
HEK293-NLRP3-INSOME cells infected with three different type-I strains and their respective isogenic mutants were used to analyze further activation of the NLRP3 inflammasome and were compared with LPS/Nigericin-treated or *S. aureus-*infected cells (**A**). The inflammasome components expression was further confirmed by Western blot in the mock control, *H. pylori*-infected, *E. coli* LPS/Nigericin-treated and *S. aureus-*infected cells (**B**). GAPDH protein immunoblots were used as a loading reference. ** *p* ≤ 0.01.

**Figure 8:**
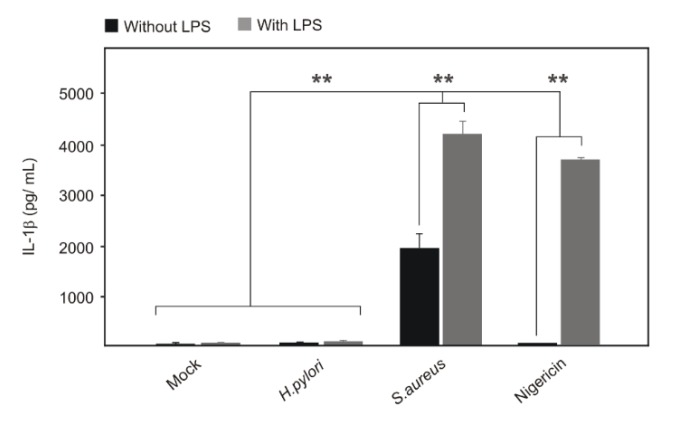
THP1 monocytes were infected with *S. aureus* and the level of mature IL-1β secretion was compared with LPS/Nigericin treatment and *H. pylori* infection. *S. aureus* infection secreted comparable levels of LPS/Nigericin treatment, which shows canonical NLPR3 inflammasome activation by this bacterial activator in monocytes and confirms the inability of *H. pylori* to activate this innate immune function. ** *p* ≤ 0.01.

As next we asked if major reported *H. pylori* virulence factors may inhibit inflammasome formation and mature IL-1β secretion in human target cells. To test this idea, three different type-I *H. pylori* wild-type strains (P12, P1 and N6) and their respective isogenic mutants of eleven important virulence factors, including *∆cagA*, *∆cagL*, *∆hopQ*, *∆hbp*, *∆ggt*, *∆tg*, *∆cgt*, *∆vacA*, *∆ureA*, *∆napA* or *∆htrA*, were applied to infect HEK293-NLRP3-INSOME cells. However, the secreted mature IL-1β concentration from *H. pylori* wild-type and isogenic mutants were not significantly different to mock-treated control cells, which ruled out the involvement of these genes in inhibiting NLRP3 inflammasome function (Figure 7A). As positive controls, *S. aureus-*infected and LPS/Nigericin-treated cells significantly upregulated the mature IL-1β secretion in HEK293-NLRP3-INSOME cells when compared to *H. pylori* infection (Figure 7A). Furthermore, the expression of all inflammasome components was confirmed in *H. pylori*- and *S. aureus-*infected. LPS/Nigericin-treated HEK293-NLRP3-INSOME cells upon Western blotting (Figure 7B; Supplementary Figure S3). Finally, *S. aureus-*infected THP1 monocytes secreted very high amounts of mature IL-1β in comparison with *H. pylori* infection, which further proves the validity of the above results (Figure 8). Thus, we identified here for the first time that *H. pylori* is unable to activate the NLRP3 inflammasome in human immune cells and secretes low amounts of IL-1β through a caspase-1 independent mechanism.

## 3. Discussion

Inflammasomes are multi-protein scaffolds formed by intracellular innate immune receptors including NLRs, AIM2 or TRIM20 for the activation of caspase-1 and cleavage of pro-forms of IL-1β and IL-18 to its mature functional molecules [57,58,59]. NLRP3 is the most studied inflammasome type and follows a two-step activation mechanism. The first step starts with the increased production of required inflammasome components for scaffold formation and activation [64,74]. The second step of NLRP3 inflammasome activation occurs by different factors discussed above, however, several research groups concluded that these factors function through key events such as K^+^ efflux, Ca^2+^ signaling, mitochondrial damage, lysosome rupture or ROS [64,74,75]. Furthermore, a new mechanism was implicated in NLRP3 activation by its recruitment to phosphatidylinositol-4-phosphate regions on the dispersed trans Golgi network through ionic bonding of conserved basic amino acid residues [76]. Certain phosphorylation events in NLRP3 also keep the molecule inactive, which were reported to be lost under chronic inflammatory conditions like CAPS (cryopyrin-associated periodic syndromes) and inflammatory bowel disease [77,78]. *H. pylori* induces the production of several cytokines including IL-1β after infection of humans or mice. There are several reports showing that *H. pylori* activated the NLRP3 inflammasome during mouse infection [39,65,66]. NLRP3 also showed non-inflammasomal function in *H. pylori* infection by maintaining the population of CD11b^+^ DCs in the gastrointestinal tissues [79]. *H. pylori* infection also impacted the NLRP3 inflammasome function in mice through MUC1, which happened through suppression of NF-κB signaling [67]. In fact, MUC1 expression in macrophages limits gastritis through regulation of the NLRP3 inflammasome [67]. However, very few studies are available on the NLRP3 inflammasome activation in human cell systems during *H. pylori* infection. In our previous report, we showed that NLRP3 protein expression is downregulated in human immune cells through miRNA-223-3p and IL-10 upon *H. pylori* infection [68]. Therefore, the present study dissected the NLRP3 inflammasome formation upon *H. pylori* infection of different human immune cells.

Human immune cells infected with different type-I and type-II *H. pylori* strains were analyzed for NLRP3 inflammasome function through the expression level of pro-IL-1β, NLRP3 inflammasome formation and secretion of mature IL-1β. Both type-I and type-II strains induced the upregulated expression of pro-IL-1β, but secreted very little mature IL-1β in THP1 monocytes after infection. In addition to the *cag*PAI, other specific virulence factors such as CagL, VacA, LPS, and urease B were also implicated in NLRP3 inflammasome activation by infected mice [39,65,66]. THP1 monocytes infected with these and other isogenic mutants of *H. pylori* virulence factors also secreted very little IL-1β, which suggests a different mechanism in human cells. As the amounts of secreted IL-1β were surprisingly very low, we compared the canonical NLRP3 inflammasome activation (*E. coli* LPS + Nigericin or ATP) with that during *H. pylori* infection. Canonical inflammasome activation yielded a high amount of mature IL-1β in comparison with *H. pylori*-infected cells. This demonstrates that our cell system is functionally intact for NLRP3 inflammasome activation, however, *H. pylori* infection failed to activate the same. There are two possibilities occurring here: (i) *H. pylori* infection lacks a proper second signal activation or (ii) irreversibly inhibits NLRP3 inflammasome formation. To test these possibilities, we treated *H. pylori*-infected cells with Nigericin or ATP, which interestingly secreted very high amounts of IL-1β and ruled out irreversible inhibition of inflammasome formation by this pathogen. Furthermore, we tried to confirm that mature IL-1β secretion is dependent on caspase-1 activation. Thus, we treated cells with VX-765, a caspase-1 inhibitor, which almost completely inhibited NLRP3 inflammasome-mediated IL-1β secretion during canonical activation in LPS-treated and *H. pylori*-infected THP1 monocytes; however, infected cells secreted very small amounts, even after caspase-1 inhibition. Conclusively, this shows that *H. pylori*-infected cells lacked a proper second signal for inflammasome activation, at least in infected THP1 monocytes, but secreted small amounts of IL-1β by a caspase-1 independent mechanism.

The very small amounts of IL-1β secretion by *H. pylori*-infected human cells can also be dependent on non-canonical NLRP3 inflammasome-mediated activation of other caspases [80,81,82]. To investigate this possibility, we infected CRISPR/Cas9 knockout THP1 monocytes for NLRP3 and caspase-1 to analyze IL-1β secretion. Either of the infected and LPS-treated knockout cell lines induced the expression of pro-IL-1β. Furthermore, *H. pylori*-infected cells secreted small amounts of IL-1β as in the parental THP1 cells. The Nigericin-induced second signal produced high amounts of mature IL-1β secretion both in infected and LPS-treated parental THP1 cells; however, NLRP3-KO and caspase-1-KO cells failed to activate NLRP3 inflammasome-mediated mature IL-1β secretion under both conditions as expected. This confirmed two scenarios: (i) the basic IL-1β secretion in *H. pylori*-infected cells is independent both of the NLRP3 inflammasome and caspase-1 activity, and (ii) in Nigericin- or ATP-treated infected cells, secretion of mature IL-1β is dependent on NLRP3 and caspase-1. Since THP-1 is an immortal cancer cell line, we next included primary human immune cells in our studies. Primary M1 and M2 macrophages were prepared from patients followed by infection with *H. pylori* for indicated time periods, which also secreted low levels of mature IL-1β as observed in parental and knockout THP-1 monocytes. However, Nigericin-treatment significantly increased mature IL-1β secretion. Thus, we speculate that *H. pylori* infection of humans in vivo might not be different to that observed in human cell systems in vitro.

The visualization of NLRP3 inflammasome formation and structure is another powerful tool to confirm the above results. We used PMA-differentiated THP1 macrophages to visualize the NLRP3 inflammasome in *H. pylori* infection and subsequent Nigericin treatment. Immuno-fluorescence microscopic pictures showed that *H. pylori*/Nigericin-treated cells revealed clear NLRP3 inflammasome speck formation, where both NLRP3 and caspase-1 co-localized in flattened elongated cells, which suggests inflammasome activation and pyroptosis. However, mock-treated control and *H. pylori*-infected THP1 macrophages maintained similar characteristic shapes and no clear signs of NLRP3 inflammasome specks. The super resolution STED microscopy for NLRP3 inflammasome speck formation in *H. pylori*/Nigericin-treated cells showed characteristic donut-shaped specks [71,76] and also secreted significantly high amounts of mature IL-1β in THP1 macrophages. Furthermore, we reconstructed the NLRP3 inflammasome by transient expression of all components including pro-IL-1β in HEK293 epithelial cells and *H. pylori* infection did not significantly change the basic mature IL-1β secretion, whereas Nigericin treatment highly increased the secretion. This finally confirmed that *H. pylori* infection of human monocytes/macrophages clearly activates the first signal but is unable to fully activate the NLRP3 inflammasome.

There are bacteria such as *S. aureus* that are known to activate the NLRP3 inflammasome [83,84,85]. We therefore compared *S. aureus* with *H. pylori* on induction of the NLRP3 inflammasome in reconstructed HEK293 cells. In concurrence with our above data, *H. pylori* infection did not change basic IL-1β secretion levels; however, *S. aureus-*infected and LPS/Nigericin-treated cells increased the reconstructed NLRP3 inflammasome activity to significantly enhance the amounts of mature IL-1β secretion. Moreover, we explored the possibility that major virulence factors of *H. pylori* could eventually inhibit the activation of the NLRP3 inflammasome. However, infection of three different wild-type strains and 11 well-known isogenic mutants did not reveal any critical effect on inflammasome function and mature IL-1β secretion. Moreover, confocal microscopy images showed increased inflammasome specks in *S. aureus-*infected and LPS/Nigericin-treated cells when compared to *H. pylori*-infected and mock control cells. Altogether, these data confirmed the inability of *H. pylori* to induce proper NLRP3 inflammasome formation in human cells. Based on the IL-1β secretion in studied cells and microscopic inflammasome analysis, it was plausible that no other inflammasome types were activated in *H. pylori*-infected cells. The increased expression of pro-IL-1β in monocytes/macrophages or other cells by *H. pylori* infection, make them vulnerable to NLRP3 inflammasome activation through increased concentration of ATP, MSU, ROS or environmental factors or co-infection with other inflammasome activating bacteria. The regulation of NLRP3 expression and activation during *H. pylori* infection may control the increased tissue destruction in the gastric mucosa and ensure bacterial survival and persistence. Together, our study created a new perspective on inflammasome manipulation by *H. pylori* and that may support chronic infection in humans. Therefore, more studies with higher animal models and patients are needed to understand the clinical perspective and possibilities for therapeutic intervention.

## 4. Materials and Methods

### 4.1. Bacterial Strains and Culture

*Helicobacter pylori* wild-type strains of type I (P1, P12, N6) and type II (UK123, UK097, Ka148) were used in this study. The P12*Δcag*A, *Δcag*L, *Δcag*PAI, *Δfla*A*, Δggt*, *Δhbp*, *Δhop*Q, *Δhp1191* (LPS), *Δcgt,* *Δtg* and *Δvac*A, as well as P1*Δnap*A, *Δure*A and *Δvac*A and N6*Δhtr*A isogenic mutants were created by insertion of a chloramphenicol or kanamycin resistance gene cassette, respectively, and were used in this study [86,87,88,89]. *H. pylori* were grown on horse serum agar plates supplemented with vancomycin (10 µg/mL), nystatin (1 µg/mL) and trimethoprim (5 µg/mL), and chloramphenicol (4 µg/mL) or kanamycin (8 µg/mL) for selection of isogenic mutants. All plates were incubated at 37 °C for 2 days in an anaerobic jar containing a campygen gas mix (Oxoid, Wesel/Germany) [88,90]. *H. pylori* grown on plates was harvested and resuspended in BHI (brain heart infusion) broth using a sterile cotton swab (Carl Roth, Karlsruhe/Germany). The bacterial concentration was measured as the optical density at 600 nm (OD_600nm_) using an Eppendorf spectrophotometer. The respective eukaryotic cells grown in medium without antibiotics and antimycotics were infected with *H. pylori* at a multiplicity of infection (MOI) of 100 [68,88]. The mock treated control cells were incubated with equal amounts of BHI broth. *Staphylococcus aureus* wild-type strain RN6390 cultured in LB broth until OD_600nm_ = 0.6–0.8 at 37 °C and 600 rpm was also used for infection [91].

### 4.2. Cell Line Cultures

THP-1 (ATCC-TIB-202) monocytic leukemia cells were cultured in RPMI-1640 medium supplemented with 10% heat-inactivated fetal bovine serum (FBS; Thermo Fisher Scientific, Dreieich/Germany), and 1% antibiotic and antimycotic solution (Sigma- Aldrich, St. Louis, MO, USA) in a humidified incubator at 37 °C with 5% CO_2_ [68,88,92]. Before infection, LPS (Sigma-Aldrich, St. Louis, MO, USA) or VX-765 inhibitor (Invivogen, Toulouse/France) treatment, cells were washed with PBS at pH 7.4 and the required number of cells were cultured in plates with antibiotic- and antimycotic-free medium. The CRISPR/Cas9 generated NLRP3 or caspase-1 knockout THP-1 cells (kind gift from Prof. Veit Hornung, Ludwig-Maximilians University, Munich, Germany) were also maintained or prepared as parental THP1 monocytes mentioned before. For macrophages, THP-1 monocytes were differentiated with 40 nM of Phorbol 12-Myristate 13-Acetate (PMA; Sigma-Aldrich, St. Louis, MO, USA) in complete RPMI-1640 medium for 48 h culture with daily replenishment of fresh medium and cells were also grown on glass cover slips for microscopy. Differentiated THP1 macrophages were rested in culture medium without PMA for another 48 h to attain the morphological and functional status of the macrophages [68,93]. Then, the cells were washed with PBS at pH 7.4 before adding antibiotic- and antimycotic-free medium for *H. pylori* infection or LPS treatment.

Human epithelial HEK293 cells (ATCC-CRL-1573) were cultured in Dulbecco’s modified Eagle medium (DMEM; Thermo Fisher Scientific, Dreieich/Germany) supplemented with 10% FBS (Thermo Fisher Scientific, Dreieich/Germany) and 1% antibiotic or antimycotic solution, respectively (Sigma-Aldrich, St. Louis, MO, USA) at 37 °C and 5% CO_2_ [88]. To increase the adherence of HEK293 cells, the used dishes were pre-coated with 0.01% poly-L-lysine (Sigma-Aldrich, St. Louis, MO, USA) at 37 °C one day prior to seeding. For transfection, 2.0 × 10^5^ cells were seeded in 12-well plates one day prior to transfection.

### 4.3. Preparation of M1 and M2 Macrophages Differentiation and Culture

PBMCs (peripheral blood mononuclear cells) were isolated by density gradient centrifugation of buffy coat preparations from the peripheral blood of volunteers (Deutsches Rotes Kreuz, Erlangen/Germany). Monocytes were isolated by adherence on plastic and cultured in the presence of 50 ng/mL GM-CSF (Berlex, USA) to generate M1 macrophages, or in the presence of 50 ng/mL M-CSF (R&D systems, Minneapolis/USA) to obtain M2 macrophages. Macrophages were detached with 1 mM EDTA (Sigma-Aldrich, St. Louis, MO, USA) solution after 6 days of culture. The phenotype was evaluated by expression of surface markers CD86, CD40, CD163 and CD204 by flow cytometry. The following antibodies were used for flow cytometry: CD163-BV421 (clone: GHI/61, BioLegend, Fell/Germany), CD40-FITC (clone: 5C3, BioLegend, Fell/Germany), CD86-PE (clone: IT2.2, BioLegend, Fell/Germany) and CD204-APC (clone: 351615, R&D systems, Minneapolis/USA).

### 4.4. NLRP3 Inflammasome Reconstruction by Transient Transfection and Expression of Components

The NLRP3 inflammasome was reconstructed in HEK293 cells with some modifications as described elsewhere [72]. We transfected 200 ng of pmCherry-C1-proIL1B, 200 ng of pEGFP-C2-NLRP3 (a gift from Christian Stehlik, Addgene plasmid # 73955; http://n2t.net/addgene:73955; RRID:Addgene_73955) [94], 20 ng of pcDNA3-Myc-ASC (a gift from Christian Stehlik, Addgene plasmid # 73952; http://n2t.net/addgene:73952; RID:Addgene_73952) [95] and 100 ng of pCI-caspase1 (a gift from Kate Fitzgerald, Addgene plasmid # 41552; http://n2t.net/addgene:41552; RRID:Addgene_41552) [96] using Lipofectamine 3000 (Thermo Fisher Scientific, Dreieich/Germany) following the manufacturer’s protocol. mCherry-pro-IL-1β was constructed by ligating the pro-IL-1β coding sequence from pCellFree_G03 IL1B (a gift from Kirill Alexandrov, Addgene plasmid # 67066; http://n2t.net/addgene:67066; RRID: Addgene_67066) [97] in-frame into a pmCherry-C1 vector backbone. Finally, the sequence was verified by sequencing. The cells were washed twice 16 h post transfection and grown in medium without antibiotics or antimycotics but supplemented with FBS.

### 4.5. SDS-PAGE and Immunoblotting

Cell lysates from harvested infected and non-infected immune cells were prepared by adding equal amounts of 2 x SDS-PAGE lysis buffer and boiling for 5 min. Polyacrylamide gels (6–12%) were used to resolve the proteins by electrophoresis, which were blotted on Immobilon-P membranes (Millipore, Massachusetts/USA) after that. The blotted membranes were blocked in TBST buffer with 5% skimmed milk or BSA for 1 hour at room temperature as described [88,92,98]. Anti-IL-1β (R&D systems, Minneapolis/USA), anti-NLRP3, anti-ASC (Adipogen, San Diego/USA), anti-caspase-1 and anti-GAPDH (Santa Cruz Biotechnology, Dallas/USA) primary antibodies were used for detection. As secondary antibodies, horseradish peroxidase-conjugated anti-goat polyvalent rabbit immunoglobulin, anti-rabbit or anti-mouse polyvalent goat immunoglobulin, respectively, were used (Thermo Fisher Scientific, Dreieich/Germany). Antibody detection was performed with the Amersham ECL Prime chemiluminescence Western blot kit (GE Healthcare, Chicago/USA) as described [88,98,99]. The band intensities were quantified using ImageLab Software 5.0 (BioRad, Hercules/USA).

### 4.6. Immunofluorescence Microscopy

The mock-treated control and infected cells were fixed with 3.8% PFA, which followed the standard protocol steps of washes, permeabilization and staining with corresponding fluorescently labelled antibodies [100]. After fixing, the cells were permeabilized with 0.25% Triton X100 for 1 min and blocked with 5% BSA in PBS for 1h. Anti-NLRP3 (Adipogen, San Diego, CA, USA) and anti-caspase-1 (Novus Biologicals, Centennial, CO, USA) were used as primary antibodies for staining and mounted with 50% vectashield (Vector Labs, Peterborough/ United Kingdom) in glycerin. FITC conjugated anti-mouse and TRITC conjugated anti-rabbit were used as secondary antibodies to visualize under the fluorescence microscope (Leica DMRE7, Leica Microsystems, Wetzlar/Germany). Separate images were taken in the corresponding channels and images were obtained by LAS AF computer software (Leica Microsystems, Wetzlar/Germany). Final images for publication were processed using ImageJ software (National Institute of Health, Bethesda/USA).

Confocal laser scanning microscopy (CSLM) of HEK293 cells was done by following this method. The cells were prepared as above and stained with DAPI (4′,6-diamidino-2-phenylindole, Roth, Germany) before being mounted with vectashield as above. These samples were studied using a Leica DMI4000B fluorescence microscope and different lasers (Leica Microsystems, Wetzlar/Germany) for visualizing DNA, eGFP-NLRP3 and mCherry-Pro-IL-1β [101]. Images were acquired by LAS AF computer software (Leica Microsystems, Wetzlar/Germany) and final images were processed as above.

### 4.7. STED Super Resolution Microscopy

The cells were processed and fixed as described above and were used for STED super resolution microscopy. The mounting agent was specially prepared as 86% glycerol consisting of 2.5% diazabicylco-2-2-2-octan (DABCO) for STED microscopy. Anti-NLRP3 (Santa Cruz, Dallas, TX/USA) antibody was used as primary antibody and a goat anti-rabbit antibody conjugated with Abberior Star Red (Abs. max 638 nm and Fluo. max 655 nm) served as the secondary antibody for visualization in the Abberior Instruments 775 STED microscope (https://www.abberior-instruments.com/products/expert-line/775-sted/) facility at Optical Imaging Centre Erlangen (OICE), Friedrich Alexander University Erlangen-Nuremberg. The samples were pulsed with 640 nm laser for initial visualization, followed by 775 nm wavelength laser for stimulated emission depletion for super resolution images, which gives the characteristic of cellular structures at nm ranges [102]. The acquired images were processed as above.

### 4.8. Quantification of Cytokines

The supernatants of *H. pylori* infected, and non-infected cells were collected and centrifuged at 12,000× *g* in a cold centrifuge at 4 °C to remove bacteria or debris before storing at −80 °C until assayed. Human IL-1β concentrations in the supernatant were determined by standard ELISA, with commercially available assay kits, described by the manufacturer (Becton Dickinson, Heidelberg/Germany).

### 4.9. Statistical Analysis

All experiments were repeated at least three times with similar results. The data were evaluated using one-way ANOVA followed by Tukey’s post hoc test with GraphPad statistical software. P values of *p* ≤ 0.05 (*), *p* ≤ 0.01 (**), *p* ≤ 0.001 (***) and *p* ≤ 0.0001 (****) were considered as statistically significant.

## 5. Conclusions

*Helicobacter pylori* is known to be associated with inflammation after colonizing the gastric mucosa. We found that *H. pylori* infection with various clinical isolates in human immune cells did not induce increased secretion of mature IL-1β secretion but rather low amounts were secreted. In mice models, *H. pylori* infection induced NLRP3 inflammasome activation and mature IL-1β secretion. However, infection in humans or cell cultures are not conclusive as described in the previous studies. We showed that NLRP3 inflammasome formation is not induced in infected human immune cells and epithelial cells expressing reconstructed inflammasome, when compared to known chemical and bacterial activators; LPS with Nigericin or ATP and *Staphylococcus aureus*, respectively. Upregulated expression and secretion of IL-1β was one of the causative factors for hyperproliferation of gastric epithelial cells and oncogenesis. Because *H. pylori* avoids NLRP3 inflammasome activation despite upregulated expression of pro-IL-1β and NLRP3 in immune cells, the pathogen slows down the host immunity through regulating massive production of mature IL-1β. This process may be crucial for bacterial colonization, survival and persistence. Externally added second signal activators such as Nigericin or ATP reversed this block, thus this gives novel options for therapeutic eradication of *H. pylori*.

## Supplementary Materials

The following are available online at https://www.mdpi.com/2072-6694/12/4/803/s1. Table S1: *H. pylori* strains and mutants used in this study.
