# Supplementary material for: Helicobacter pylori Avoids the Critical Activation of NLRP3 Inflammasome-Mediated Production of Oncogenic Mature IL-1β in Human Immune Cells
Source: Cancers (Basel). 2020 Mar 27;12(4):803. doi: 10.3390/cancers12040803 (PMC7226495; doi:10.3390/cancers12040803)
Supplement: Supplementary file 1 [file cancers-12-00803-s001.pdf]

Supplementary Materials

Helicobacter pylori Avoids the Critical Activation of NLRP3 Inflammasome-Mediated Production of Oncogenic Mature IL-1β in Human Immune Cells

Suneesh Kumar Pachathundikandi, Nicole Blaser, Heiko Bruns and Steffen Backert

Table 1. *H. pylori* wild-type strains and mutants used in this study.

Name	Gene	Function	Resistance	Reference
P1	wt	*cag*PAI+ type-I strain	None	[1]
P12	wt	*cag*PAI+ type-I strain	None	[2]
N6	wt	*cag*PAI+ type-I strain	None	[3]
UK123	wt	*cag*PAI- type-II strain	None	M. Achtman & S. Suerbaum (unpublished)
UK097	wt	*cag*PAI- type-II strain	None	M. Achtman & S. Suerbaum (unpublished)
Ka148	wt	*cag*PAI- type-II strain	None	[4]
P1Δ*nap*A	*napA*	Neutrophil activating protein A	Chl	[5]
P1Δ*ure*A	*ureA*	Urease subunit A	Chl	This study
P1Δ*vac*A	*vacA*	Vacuolating cytotoxin A	Kan	[6]
P12Δ*cag*A	*cagA*	T4SS effector protein	Chl	[7]
P12Δ*cag*L	*cagL*	T4SS tip protein, integrin binding	Chl	[8]
P12Δ*cag*PAI	*cag*PAI	Entire T4SS apparatus and effector protein CagA	Kan	[9]
P12Δ*vac*A	*vacA*	Vacoulating cytotoxin A	Chl	[6]
P12Δ*fla*A	*flaA*	Flagella monomer		[7]
P12Δ*ggt*	*ggt*	γ-glutamyl transferase	Chl	This study
P12Δ*hbp*	*hbp*	Phosphoheptose isomerase	Chl	This study
P12Δ*hopQ*	*hopQ*	Outer membrane protein and adhesin	Chl	[10]
P12Δ*1191*	*Hp1191*	Glycosyl-transferase	Kan	[11]
P12Δ*cgt*	*cgt*	Cholesterol-glycosyl transferase	Kan	This study
P12Δ*slt*	*slt*	Lytic murein transglycosylase	Chl	This study
N6Δ*htr*A	*htrA*	Secreted serine protease	Kan	[12]

Abbreviations: Chl: Chloramphenicol, Kan: Kanamycin, wt: Wild-type

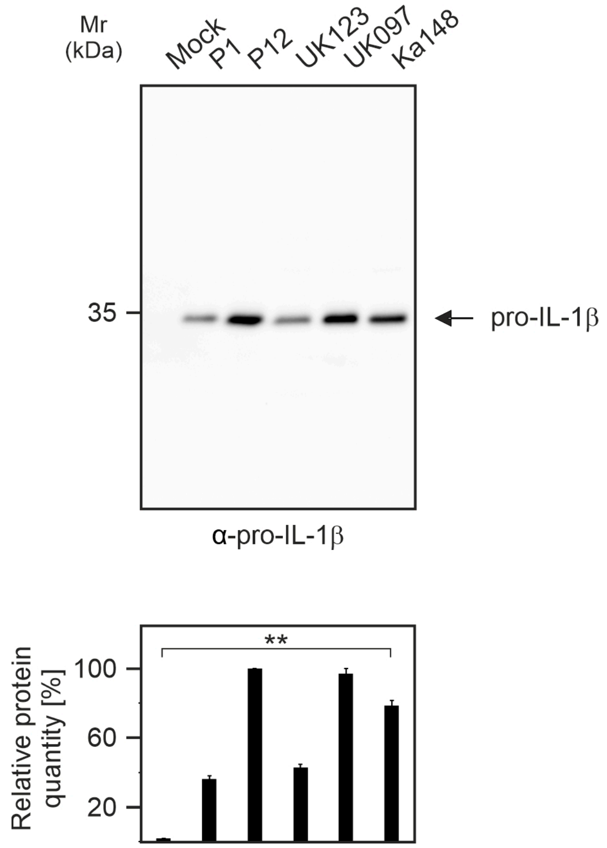

Figure S1: Full-size blot of Figure 1A and quantification of pro-IL-1β protein expression in *H. pylori*-infected THP1 monocytes. Shown is the blot of Figure 1A detecting pro-IL-1β using a specific antibody against pro-IL-1β. The band intensities were quantified using ImageLab Software version 5.0 and shown as relative intensities. The strongest band was set as 100 %. $^{**}p \leq 0.01$

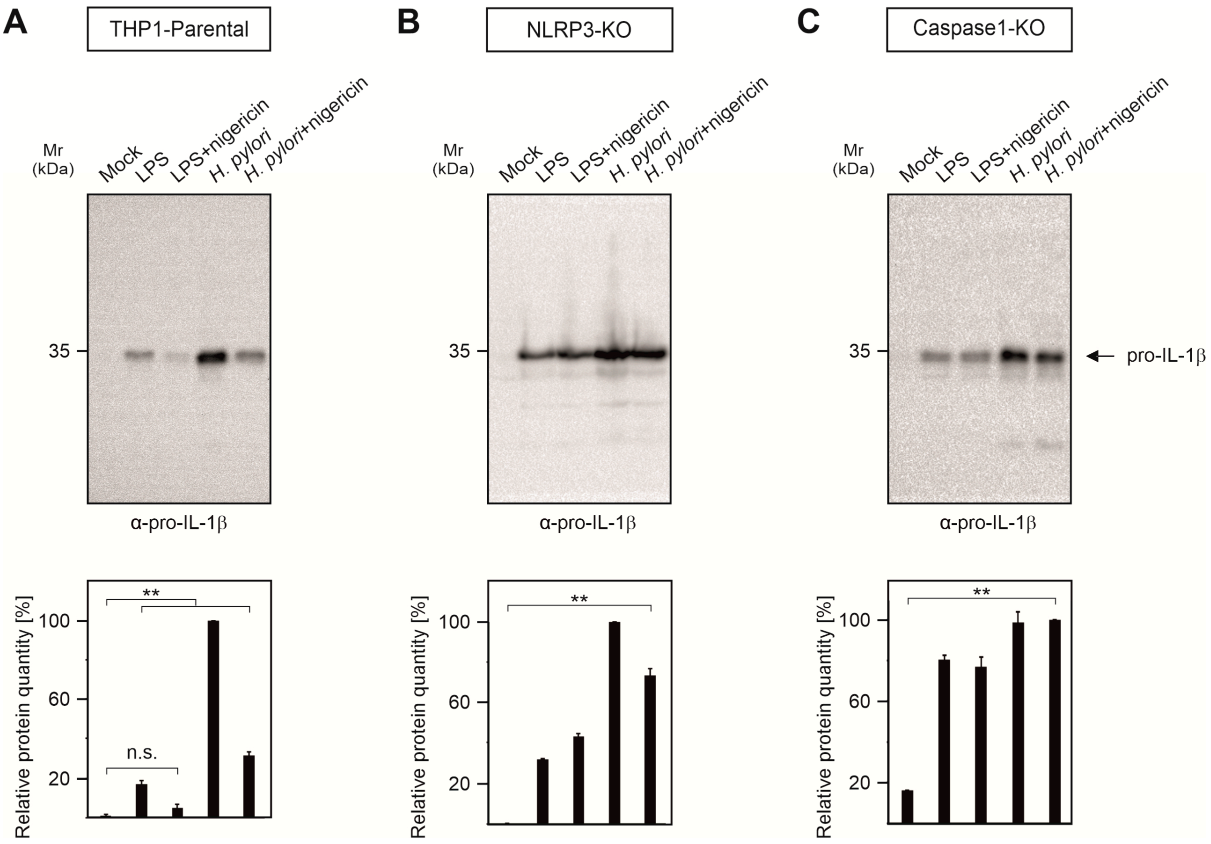

Figure S2: Full-size blots of Figure 3A and quantification of pro-IL-1β protein expression in THP1 parental **(A)**, THP1 NLRP3-KO **(B)** and THP1 Caspase-1-KO **(C)** monocytes. After treatment of the cells with *E. coli* LPS, *H. pylori* infection and NLRP3 inflammasome activation by nigericin, pro-IL-1β protein expression was detected by Western blotting using a specific antibody. Using ImageLab Software version 5.0 the band intensities of pro-IL-1β were quantified and relative amounts are displayed. The strongest band in each blot was set as 100 %. ** $p \leq 0.01$

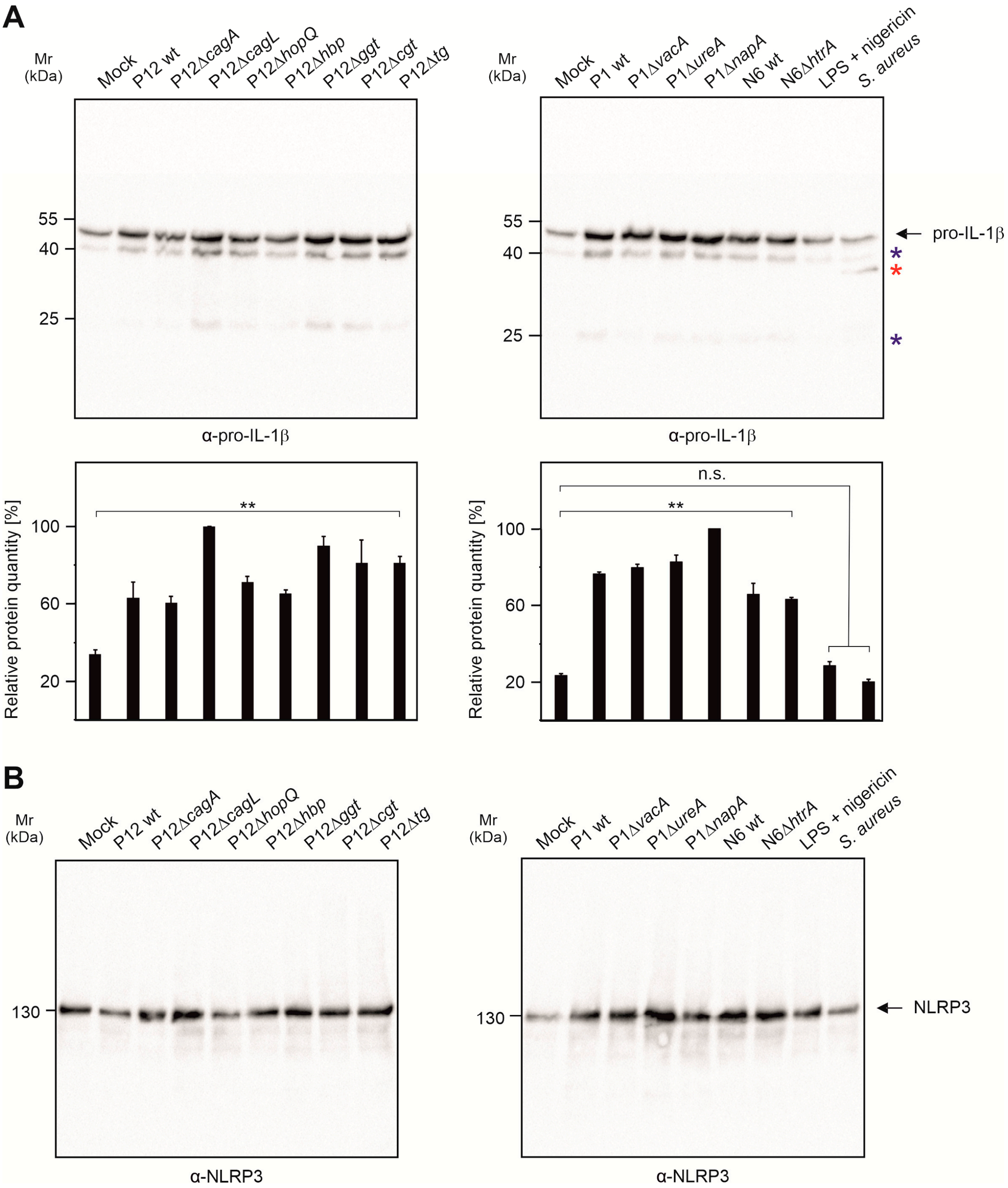

Figure S3: To be continued.

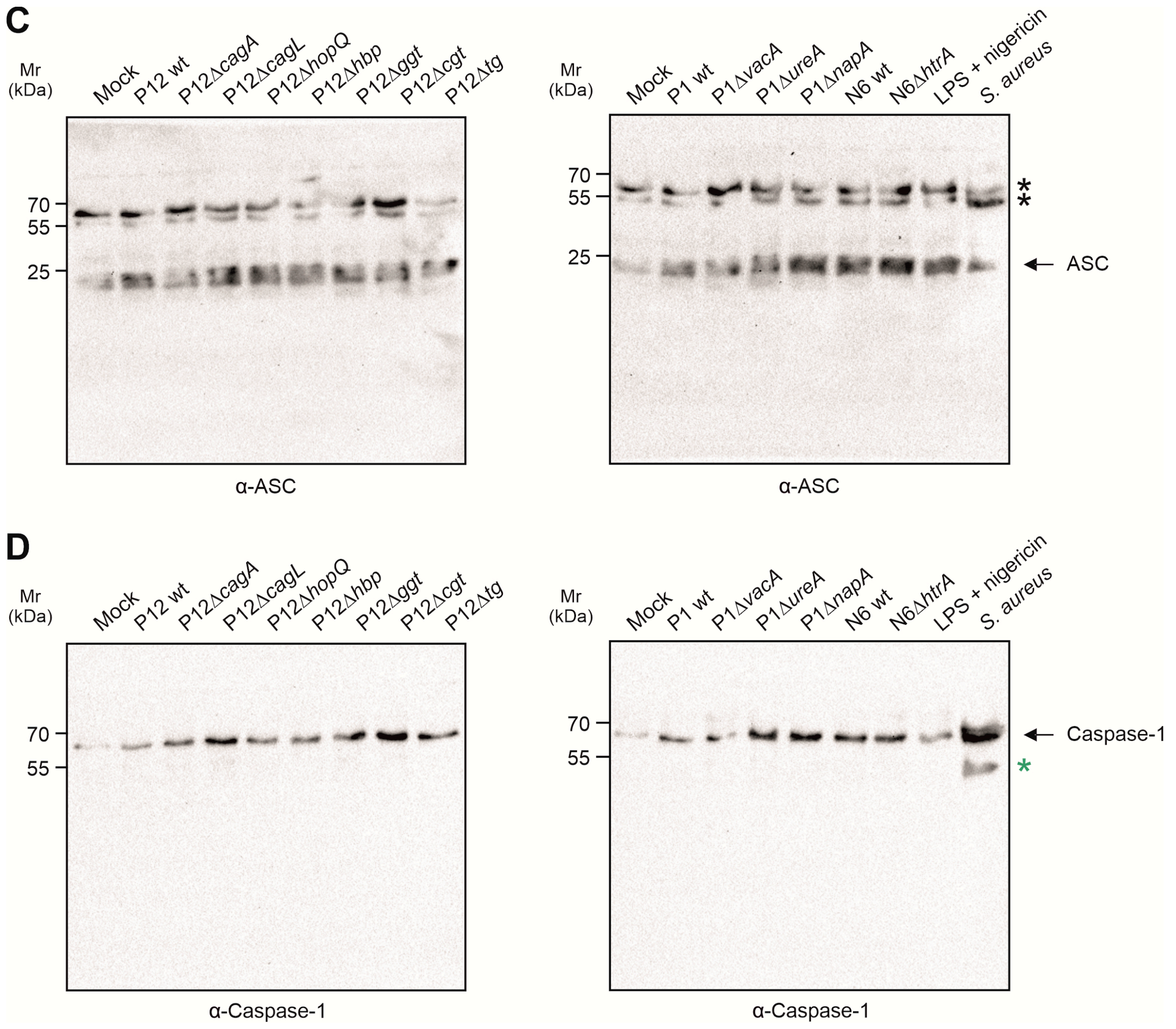

Figure S3: Full-size blots of Figure 7 and quantification of pro-IL-1β protein expression in *H. pylori*-infected HEK293-NLRP3-INSOME cells. These cells were infected with *H. pylori* deletion variants or the corresponding wt strains. In addition, these cells were treated with *E. coli* LPS and nigericin or infected with *S. aureus*. Using Western blotting the pro-IL-1β protein expression was detected by a specific antibody **(A)**. A *S. aureus*-specific cleavage product of IL-1β was detected (red asterisk). Additionally, cross-reaction of the antibody led to bands of unknown origin with a molecular weight of 40 kDa and 25 kDa (blue asterisk). The band intensities of pro-IL-1β were quantified using ImageLab Software version 5.0 and shown as relative amounts. The strongest band was set as 100 %. As control, the protein expression of NLRP3 **(B)**, ASC **(C)** and Caspase-1 **(D)** were investigated using specific antibodies. The antibody against ASC showed some cross-reactivity leading to a double band of unknown origin having a molecular weight between 55 kDa and 70 kDa (black asterisks). Detection of caspase-1 revealed a *S. aureus*-specific band with a molecular weight of 55 kDa (green asterisk). ** $p \leq 0.01$

References

1. Moese, S.; Selbach M.; Zimny-Arndt, U.; Jungblut P.R.; Meyer, T.F.; Backert, S. Identification of a tyrosine-phosphorylated 35 kDa carboxy-terminal fragment (p35CagA) of the *Helicobacter pylori* CagA protein in phagocytic cells: Processing or breakage? *Proteomics* **2001**, 1, 618–629.
2. Fischer, W.; Windhager L.; Rohrer, S.; Zeiller, M.; Karnholz, A.; Hoffmann, R.; Zimmer, R.; Haas, R. Strain-specific genes of *Helicobacter pylori*: genome evolution driven by a novel type IV secretion system and genomic island transfer. *Nucleic Acids Res.* **2010**, 38, 6089–6101.
3. Ferrero, R.L.; Cussac, V., Courcoux, P.; Labigne, A. Construction of isogenic urease-negative mutants of *Helicobacter pylori* by allelic exchange. *J. Bacteriol.* **1992**, 174, 4212–4217.
4. Backert, S.; Schwarz, T.; Miehlke, S.; Kirsch, C.; Sommer, C.; Kwok, T.; Gerhard, M.; Goebel, U.B.; Lehn, N.; Koenig, W.; Meyer, T.F. Functional analysis of the cag pathogenicity island in *Helicobacter pylori* isolates from patients with gastritis, peptic ulcer, and gastric cancer. *Infect. Immun.* **2004**, 72, 1043–56.
5. Brisslert, M.; Enarsson, K.; Lundin, S.; Karlsson, A.; Kusters, J.G.; Svennerholm, A.M.; Backert, S.; Quiding-Järbrink, M. *Helicobacter pylori* induce neutrophil transendothelial migration: role of the bacterial HP-NAP. *FEMS Microbiol Lett.* **2005**, 249(1), 95-103.
6. Tegtmeyer, N.; Harrer, A.; Schmitt, V.; Singer, B.B.; Backert, S. Expression of CEACAM1 or CEACAM5 in AZ-521 cells restores the type IV secretion deficiency for translocation of CagA by *Helicobacter pylori*. *Cell Microbiol.* **2019**, 21(1), e12965.
7. Pachathundikandi, S.K.; Tegtmeyer, N.; Arnold, I.C.; Lind, J.; Neddermann, M.; Falkeis-Veits, C.; Chattopadhyay, S.; Brönstrup, M.; Tegge, W.; Hong, M.; Sticht, H.; Vieth, M.; Müller, A.; Backert, S. T4SS-dependent TLR5 activation by *Helicobacter pylori* infection. *Nat Commun.* **2019**, 10(1), 5717.
8. Kwok, T.; Zabler, D.; Urman, S.; Rohde, M.; Hartig, R.; Wessler, S.; Misselwitz, R.; Berger, J.; Sewald, N.; König, W.; Backert, S. *Helicobacter* exploits integrin for type IV secretion and kinase activation. *Nature*. **2007**, 449(7164), 862-6.
9. Backert, S.; Kwok, T.; Schmid, M.; Selbach, M.; Moese, S.; Peek, R.M. Jr; König, W.; Meyer, T.F.; Jungblut, P.R. Subproteomes of soluble and structure-bound *Helicobacter pylori* proteins analyzed by two-dimensional gel electrophoresis and mass spectrometry. *Proteomics*. **2005**, 5(5), 1331-45.
10. Moonens, K.; Hamway, Y.; Neddermann, M.; Reschke, M.; Tegtmeyer, N.; Kruse, T.; Kammerer, R.; Mejías-Luque, R.; Singer, B.B.; Backert, S.; Gerhard, M.; Remaut, H. *Helicobacter pylori* adhesin HopQ disrupts *trans* dimerization in human CEACAMs. *EMBO J*. **2018**,37(13).
11. Dolan, B.; Naughton, J.; Tegtmeyer, N.; May, F.E.; Clyne, M. The interaction of *Helicobacter pylori* with the adherent mucus gel layer secreted by polarized HT29-MTX-E12 cells. *PLoS One*. **2012**, 7(10), e47300.
12. Zawilak-Pawlik, A.; Zarzecka, U.; Żyła-Uklejewicz, D.; Lach, J.; Strapagiel, D.; Tegtmeyer, N.; Böhm, M.; Backert, S.; Skorko-Glonek, J. Establishment of serine protease htrA mutants in *Helicobacter pylori* is associated with secA mutations. *Sci Rep*. **2019**, 9, 11794.
